# Comparison of Machine Learning Algorithms for Predicting Spine Surgery Duration

**DOI:** 10.3390/medicina62071308

**Published:** 2026-07-06

**Authors:** Myungjin Ko, Hyung Chul Lee, Hyun Seong Lee, Byeongcheol Lee, Min Woo Lee, Yu Jeong Kim, Jae Hong Park

**Affiliations:** 1 Department of Anesthesiology and Pain Medicine, Inje University Haeundae Paik Hospital, Busan 48108, Republic of Korea; h00319@paik.ac.kr (M.K.);; 2 Department of Anesthesiology and Pain Medicine, Seoul National University College of Medicine, Seoul National University Hospital, Seoul 03080, Republic of Korea; vital@snu.ac.kr; 3 Healthcare AI Research Institute, Seoul National University Hospital, Seoul 03080, Republic of Korea

**Keywords:** machine learning, predictive analytics, operating room management, resource allocation, explainable artificial intelligence

## Abstract

*Background and Objectives*: Accurate prediction of surgical duration is essential for efficient operating room management. Spine surgery frequently shows discrepancies between estimated and actual surgical duration, which can disrupt surgical scheduling and resource allocation. The aim of this study was to develop and compare machine learning (ML) algorithms for predicting spine surgery duration and identify the most effective approach. *Materials and Methods*: Electronic medical records of 3376 patients who underwent spine surgery were retrospectively analyzed at a single center. The dataset was divided into training (80%, *n* = 2700) and internal test (20%, *n* = 676) sets using stratified random sampling based on surgical duration quintiles. To match the intended use at the time of operating room scheduling, four models (Random Forest, XGBoost, multilayer perceptron [MLP], and weighted least squares [WLS] regression) were developed using only predictors available at scheduling and evaluated on the independent internal test set; a full-information model that additionally included intraoperatively recorded variables was examined for comparison. *Results*: XGBoost demonstrated the best predictive performance, achieving a mean squared error (MSE) of 3014.6 min^2^ (equivalent to a root mean squared error [RMSE] of 54.9 min; 95% CI for MSE, 2558.3–3556.0) and an R^2^ of 0.622 (95% CI, 0.566–0.675). The other ML models showed comparable performance, whereas WLS regression performed less favorably; the full-information model performed equivalently (R^2^ 0.622). Compared with surgeon-estimated duration alone, XGBoost reduced mean absolute error by approximately 20 min (paired ΔMAE −20.1 min; 95% CI, −23.7 to −16.5) and improved prediction accuracy within 60 min by 14.0 percentage points. SHapley Additive exPlanations (SHAP) analysis identified surgeon identity as the most influential predictor across all models (24.7–41.9%), followed by procedure type and surgeon-estimated duration. *Conclusions*: Machine learning models substantially improved prediction of spine surgery duration compared with conventional approaches, with XGBoost showing the highest predictive accuracy. Surgeon identity emerged as the most important predictor of surgical duration. Implementation of such models may improve operating room scheduling efficiency and resource allocation but requires prospective evaluation of clinical and workflow outcomes.

## 1. Introduction

Accurate prediction of surgical duration is a key element in efficient operating room scheduling and resource management [1,2]. In spine surgery, discrepancies between actual and planned operative times frequently occur, resulting in scheduling inefficiencies [3]. Inaccurate predictions reduce operating room utilization, cause unnecessary waiting for medical staff, and may lead to surgical delays, cancellations, and decreased patient satisfaction [4]. In addition, inaccurate prediction of surgical duration may increase overtime costs, staffing inefficiencies, and underutilization of operating room resources, resulting in a substantial economic burden on healthcare systems. Therefore, improving the accuracy of surgical duration prediction is important not only for workflow optimization but also for cost-effective operating room management [5].

Traditionally, surgical duration prediction has relied on surgeons’ subjective experience or historical averages of similar procedures. These approaches have clear limitations in systematically accounting for surgical complexity, patient characteristics, and surgeon experience [6,7]. Recent advances in machine learning (ML) have provided opportunities to learn complex nonlinear patterns from large medical datasets and improve prediction accuracy [8,9]. In spine surgery, ML techniques have increasingly been applied to diagnosis, outcome prediction, cost estimation, and surgical decision-making, demonstrating their growing role across the spectrum of spine care [10,11]. Tree-based ensemble models such as Random Forest [12] and XGBoost [13] have demonstrated strong predictive performance in medical applications, and ensemble approaches have been applied to spine surgery duration prediction [14]. ML-based prediction has also been successfully applied to other clinical outcomes, including surgical site infection and postoperative complications [15,16].

Despite their strong performance, complex ML models are often perceived as “black boxes,” and clinicians may hesitate to adopt models whose decision-making processes remain opaque [17]. SHAP (SHapley Additive exPlanations), a game theory-based method, addresses this limitation by quantifying how each predictor contributes to individual predictions, thereby improving transparency and facilitating clinical trust [18,19].

Equally critical for reliable model development is a rigorous validation framework. Models evaluated using data involved in the training process may show inflated performance that fails to generalize to new clinical settings [20]. Evaluation using an independent test dataset, strictly separated from all model development steps including feature engineering and preprocessing, is essential for unbiased assessment of generalizability.

This study compared four approaches—Random Forest, XGBoost, multilayer perceptron (MLP), and weighted least squares (WLS) regression—for predicting spine surgery duration. Models were developed using 5-fold cross-validation and evaluated on an independent test set. SHAP analysis was applied to all models to improve interpretability. The primary aim was to compare the predictive accuracy of four machine-learning approaches—Random Forest, XGBoost, MLP, and WLS regression—for spine surgery duration under a rigorous internal-validation framework, benchmarked against the surgeon’s own preoperative estimate. The secondary aim was to identify the most influential predictors of operative time using SHAP analysis applied uniformly across all models. Although ensemble models with SHAP-based interpretation have previously been reported for spine surgery duration [14], the present study is distinguished by an explicitly deployment-oriented design in which the primary model is restricted to information available at the time of operating-room scheduling.

## 2. Methods

### 2.1. Data Collection and Preprocessing

#### 2.1.1. Study Population and Data Source

This retrospective study analyzed electronic medical records of patients who underwent spine surgery at Inje University Haeundae Paik Hospital from 15 March 2010 through 31 December 2020. The study protocol was approved by the Institutional Review Board of Haeundae Paik Hospital (HPIRB) (approval number: HPIRB 2025-05-015; approval date: 26 May 2025), and the requirement for informed consent was waived by the Institutional Review Board because this retrospective study involved analysis of existing de-identified clinical data and posed minimal risk to participants. Spine surgery cases were identified from the orthopedic electronic medical record (EMR) database using procedure names containing the keywords “Spine,” “Laminoplasty,” “Diskectomy,” and “Laminectomy.” The inclusion criterion was any spine surgery performed at the institution during the study period and identified by the keywords above. The exclusion criterion was a procedure type performed fewer than 50 times over the study period (rare procedures), excluded because the limited sample size was insufficient for reliable model development and evaluation. No other inclusion or exclusion criteria were applied. During the study period, 3723 spine surgeries were identified, of which 347 (9.3%) were excluded by this criterion, leaving 3376 cases for the final analysis. No additional cases were excluded for missing operative duration, implausible timestamps, duplicated or combined procedures, or incomplete records. The cohort selection process is summarized in Supplementary Figure S1.

The dataset included the following variables: surgical procedure name (Op_name), diagnosis (Diagnosis), surgeon-estimated surgical duration (duration_expected), actual surgical duration (duration_actual), surgeon (Surgeon), anesthesia start time (Start), year (Year), month (Month), anesthesiologist (Staff), anesthesia resident (Resident), and number of involved spinal levels (level_count). During preprocessing, the “Start” variable was converted into minutes from midnight to create a new feature (Start_minutes) in order to account for potential time-of-day effects. Categorical variables including surgical procedure name (Op_name), diagnosis (Diagnosis), surgeon identity (Surgeon), anesthesiologist (Staff), anesthesia resident (Resident), sex, and emergency status were encoded using one-hot encoding, generating binary indicator variables for each category. Binary features with fewer than 10 positive cases (representing less than 0.3% of total samples) were removed to reduce dimensionality and prevent overfitting to rare categories. After this preprocessing, 133 predictor variables were generated. The primary (scheduling-time) models used the 90 predictors available at the time of operating room scheduling; a full-information model that additionally included intraoperatively recorded variables (133 predictors) was evaluated for comparison (Section 2.1.1, prediction time point). The complete list of predictor variables, with each variable’s availability at the time of scheduling, is provided in Supplementary Table S3.

The intended prediction time point is the point of operating room scheduling, at which the procedure, working diagnosis, primary surgeon, surgeon-estimated duration, and patient characteristics are available, whereas staffing assignments and intraoperative details are not. Accordingly, the primary models were built using only the 90 predictors available at the time of scheduling; variables that are not known at scheduling (anesthesia start time, anesthesiologist and resident assignment, anesthetic agents, and anesthesia method) were therefore excluded from the primary models. For comparison, a full-information model that additionally included these intraoperatively recorded variables (133 predictors) was also developed; its performance relative to the primary model is reported in Section 3.1 and Supplementary Table S2.

To ensure robust evaluation and avoid data leakage [20], the dataset was divided into training and test sets using stratified random sampling based on surgical duration quintiles. The training dataset consisted of 80% of the data (*n* = 2700), and the remaining 20% (*n* = 676) was reserved as an independent test dataset. Missing values were handled in two stages: KNN imputation (k = 5) of a small number of low-missingness baseline variables (weight, hematocrit, ASA score, and anesthesiologist information; each <5% missing) was applied to the full dataset before the split, and after the split, any remaining missing numerical values were imputed using the median of each feature calculated from the training set only. One-hot category learning, sparse-feature filtering, and scaling were likewise fitted on the training set only, so the test set was excluded from model fitting and from all training-derived preprocessing. The potential effect of the pre-split KNN step on test performance was examined in a sensitivity analysis (Supplementary Table S2) and is addressed in the Limitations.

#### 2.1.2. Variable Extraction and Processing

Key clinical variables were extracted from unstructured text data in anesthesia records and inpatient medical records. From anesthesia records, the “blood order history” section was analyzed using the Levenshtein distance algorithm to extract transfusion (blood-product) information (packed RBC, fresh frozen plasma, and platelet concentrate). These transfusion variables were deliberately excluded from the models: transfusion is recorded intraoperatively and is strongly associated with operative difficulty and duration, so using it as a predictor would be inconsistent with the study aim of prediction from preoperatively available information. Binary indicators of which anesthetic agents were used (sevoflurane, desflurane, oxygen, and remifentanil) were retained; because these largely duplicate the recorded anesthesia method and are determined intraoperatively, they carry little independent information. Like the other non-preoperative variables, they are included only in the full-information comparison model and are excluded from the primary scheduling-time models; their effect on predictive performance is reported in Section 3.1 and Supplementary Table S2. The predictors used in the models are listed in Supplementary Table S3. From inpatient records, regular expressions were used to separately extract the “Diagnosis (Impression)” and “Treatment Plan (Plan)” sections. These data were used to identify the spinal levels involved in surgery (e.g., L3–4–5) and to calculate the number of involved levels.

To protect surgeon privacy, surgeon names were replaced with alphabetical codes (A, B, C, etc.). Sex was binarized (male = 1, female = 0), and American Society of Anesthesiologists (ASA) physical status was standardized as integer values ranging from 1 to 6. Surgical duration and estimated duration were converted from hour–minute format to integer values in minutes. Body mass index (BMI) was calculated using body weight and height.

#### 2.1.3. Handling of Missing Values

The proportion of missing values for the main continuous variables (weight, hematocrit, and ASA score) was low (<5% of observations for each variable). Prior to the train-test split, these missing values were handled using the k-nearest neighbors (KNN) imputation method (k = 5). Missing weight values were imputed using age, sex, height, and diabetes status. Missing hematocrit values were imputed using age and sex. Missing ASA scores were imputed using information on emergency status, age, diabetes, hypertension, heart disease, and other comorbidities. Missing hemoglobin values were estimated by dividing the imputed hematocrit values by 3. Missing values for anesthesiologist information (specialist/resident) were imputed using the KNN method after one-hot encoding transformation, with additional adjustment based on yearly frequency distributions. After the train-test split, any remaining missing numerical values were imputed using the median of each feature calculated exclusively from the training data, ensuring no information from the test set influenced the imputation process.

### 2.2. Model Development

Four predictive models were developed to estimate surgical duration: Random Forest [12], XGBoost [13], MLP, and WLS regression. Hyperparameter optimization was performed using grid search with 5-fold cross-validation on the training dataset. In each fold, the model was trained on 80% of the training data and validated on the remaining 20%. This procedure ensured that each data point was used in both training and validation, providing a robust estimate of model performance. Candidate ranges for key hyperparameters, including the number of estimators, tree depth, learning rate, regularization parameters, and neural network architecture, were explored for each model. The final model configuration was selected based on cross-validation performance. Unless otherwise specified, reported models use the scheduling-time predictor set; an otherwise identical full-information model (same algorithms, hyperparameters, and preprocessing, applied to all 133 predictors) is reported for comparison.

The selected final hyperparameters were as follows. XGBoost: 250 estimators, maximum tree depth 7, learning rate 0.03, subsample 0.5, column subsample 0.5, gamma 0.2, L2 regularization (λ) 1.0, and L1 regularization (α) 0.5. Random Forest: 200 trees, maximum depth 50, minimum samples per split 8, minimum samples per leaf 1, maximum features 0.3, with bootstrap aggregation. MLP: two hidden layers (100 and 50 neurons), ReLU activation, Adam optimizer, L2 penalty (α) 0.01, constant learning rate (initial 0.001), input features standardized to zero mean and unit variance, and early stopping; the remaining settings followed scikit-learn defaults. The complete search spaces and selected values are provided in Supplementary Table S1.

The weighted least squares (WLS) regression model was included as a transparent linear comparator. The target variable was Box–Cox transformed (λ = −0.10) to address skewness, and residual outliers were removed from the training data using the 1.5 × IQR rule. Lasso (L1, α = 0.01) was then applied for automatic feature selection (12–13 features retained per fold), and a weighted least squares model was fitted on the selected features, with weights set to the inverse of the squared ordinary-least-squares residuals to correct heteroscedasticity. All predictions were back-transformed to the original minute scale before performance metrics were computed. Detailed hyperparameter search ranges for each model are provided in Supplementary Table S1.

### 2.3. Model Evaluation and Analysis

Model performance was evaluated using an independent test dataset (*n* = 676) that was not used during model development. This strict separation ensured an unbiased evaluation of model generalizability. Primary performance metrics included mean squared error (MSE, expressed in min^2^) and the coefficient of determination (R^2^). Additional metrics included root mean squared error (RMSE), mean absolute error (MAE), and median absolute error (MedAE), all expressed in minutes. To estimate statistical uncertainty, bootstrap resampling with 1000 iterations was used to calculate 95% confidence intervals for all metrics. In addition, paired bootstrap resampling (5000 iterations over the common set of test cases) was used to estimate 95% confidence intervals for the differences between models, and between each model and the surgeon-estimate baseline, in MAE, RMSE, R^2^, and accuracy within ±30 and ±60 min.

To further improve model interpretability, SHAP (SHapley Additive exPlanations) analysis [18] was implemented for all models. For tree-based models (Random Forest and XGBoost), the exact TreeExplainer algorithm was used to compute SHAP values efficiently and without approximation error [18]. For the MLP and WLS models, the KernelExplainer framework was applied with 500 perturbation samples to approximate SHAP values. Background instances were sampled from the training dataset (*n* = 300) to represent the reference distribution, and SHAP values were computed for 300 test instances. The analysis quantified the contribution of individual features to model predictions and enabled assessment of both individual feature importance and grouped feature importance. Grouped feature importance was computed by summing the mean absolute SHAP values of all one-hot indicator variables belonging to each original categorical predictor (for example, the nine surgeon indicators) and normalizing these values across predictors to sum to 100%. Restricting this analysis to 300 test instances limited the computational cost of KernelExplainer, and the stability of the resulting grouped-importance estimates under repeated resampling is reported in Supplementary Table S2. Prediction error analysis was also conducted by examining the distribution of prediction errors across different ranges of surgical duration.

## 3. Results

### 3.1. Model Performance Comparison

Four machine learning models (Random Forest, XGBoost, MLP, and WLS regression) were evaluated to predict spine surgery duration using the predictors available at the time of operating room scheduling. Table 1 summarizes the performance metrics on the independent internal test dataset (*n* = 676). The XGBoost model demonstrated the best performance across all metrics, achieving the lowest MSE of 3014.6 min^2^ (RMSE 54.9 min; 95% CI for MSE, 2558.3–3556.0) and the highest R^2^ of 0.622 (95% CI, 0.566–0.675). The MLP performed comparably (MSE 3021.1 [95% CI, 2544.5–3536.1]; R^2^ 0.621 [95% CI, 0.563–0.671]), followed closely by Random Forest (MSE 3106.2 [95% CI, 2618.9–3655.3]; R^2^ 0.610 [95% CI, 0.555–0.660]). WLS regression showed the lowest performance among the four models (MSE 3616.7 [95% CI, 2984.1–4322.3]; R^2^ 0.546 [95% CI, 0.480–0.606]).

The MSE confidence intervals of the three ML models (XGBoost, MLP, and Random Forest) substantially overlapped, suggesting that performance differences among these models were small in practical terms. MSE differences among them ranged from 6.5 (XGBoost vs. MLP) to 91.6 (XGBoost vs. Random Forest). All three ML models outperformed WLS regression, with MSE differences ranging from 510.5 to 602.1.

In paired bootstrap comparisons on the common test cases, XGBoost significantly outperformed the surgeon-estimate baseline (assessed on the 613 test cases with a recorded estimate) across all metrics (ΔMAE −20.1 min, 95% CI −23.7 to −16.5; ΔRMSE −27.6 min, 95% CI −32.0 to −23.1; ΔR^2^ +0.48, 95% CI +0.40 to +0.56; accuracy within ±60 min +14.0 percentage points, 95% CI +10.1 to +18.3). Differences between XGBoost and the next-best models were small and not statistically significant (versus MLP: ΔMAE −0.7 min, 95% CI −2.6 to +1.2; ΔR^2^ +0.00, 95% CI −0.04 to +0.04; versus Random Forest: ΔMAE −0.7 min, 95% CI −1.8 to +0.4; ΔR^2^ +0.01, 95% CI −0.01 to +0.03), whereas XGBoost significantly outperformed WLS (ΔMAE −3.2 min, 95% CI −5.3 to −1.2; ΔR^2^ +0.08, 95% CI +0.03 to +0.12). Full paired comparisons are provided in Supplementary Table S2. For comparison, a full-information model that additionally included intraoperatively recorded variables (anesthesia staffing, anesthesia method, anesthetic agents, and start time; 133 predictors) achieved equivalent performance (XGBoost R^2^ 0.622; MAE 39.1 min), confirming that restricting the model to scheduling-time predictors did not reduce accuracy. Cross-validation and independent-test performance were closely aligned across models. The difference between mean 5-fold cross-validation R^2^ and independent-test R^2^ was small (+0.039 for XGBoost, +0.037 for Random Forest, +0.084 for MLP, and +0.079 for WLS), suggesting no evidence of substantial overfitting despite the relatively high-dimensional feature space. Sensitivity analyses showed that varying the sparse-feature threshold from 1 to 20 positive cases changed test-set R^2^ by less than approximately 0.02, indicating that model performance was not dependent on the specific threshold selected (Supplementary Table S2).

### 3.2. Performance Improvement Compared with Baseline Prediction

To quantify the practical improvement achieved by machine learning models, the baseline prediction based solely on surgeon-estimated duration (duration_expected) was evaluated. Table 2 summarizes the performance improvement achieved by machine learning models over the baseline prediction across key metrics. Among the 613 test cases with a recorded surgeon estimate (the remaining 63 cases [9.3%] had no preoperative estimate), the baseline approach showed limited predictive performance, with an MSE of 6964.8 min^2^, an MAE of 59.8 min, and an R^2^ of 0.140, indicating that surgeon-estimated duration alone explained approximately 14.0% of the variability in actual operative time. The baseline also showed a systematic underestimation bias, with the estimate being on average 44.6 min shorter than the actual operative time. Notably, the machine-learning models generated a prediction for every case, including the 63 (9.3%) for which no surgeon estimate was available.

Compared with this baseline, machine learning models substantially improved predictive performance. The coefficient of determination increased approximately 3.9–4.4-fold relative to baseline. The XGBoost model improved R^2^ from 0.140 to 0.622, corresponding to a 344% increase in explained variance. Prediction accuracy within 60 min improved from 65.9% to approximately 76–80% across models, and accuracy within 30 min from 39.5% to approximately 49–54%. On the common test cases, mean absolute error decreased by approximately 20 min (paired ΔMAE −20.1 min, 95% CI −23.7 to −16.5; XGBoost MAE 39.2 min), a relative reduction of roughly one-third.

Accuracy within 30 or 60 min indicates the proportion of cases in which the predicted duration fell within ±30 or ±60 min of the actual operative duration.

### 3.3. Prediction Error Analysis

**Figure 1:**
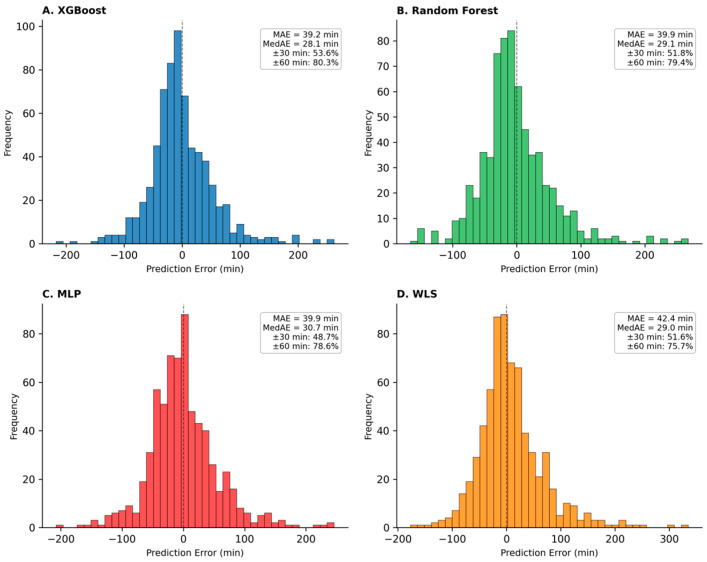
Distribution of prediction errors (actual duration—predicted duration) for each machine learning model. (**A**) XGBoost, (**B**) Random Forest, (**C**) MLP, (**D**) WLS regression. The dashed vertical line indicates zero error. Inset boxes show MAE, MedAE, and prediction accuracy within ±30 and ±60 min.

Figure 1 shows the distribution of prediction errors (actual duration—predicted duration) for each model. All models exhibited slightly right-skewed error distributions, indicating a tendency to underestimate surgical duration for procedures that actually required longer operative times. The XGBoost model showed the narrowest error distribution. In the analysis of prediction accuracy within 30 min, XGBoost achieved 53.6%, Random Forest 51.8%, MLP 48.7%, and WLS 51.6%. For prediction accuracy within 60 min, the corresponding values were 80.3%, 79.4%, 78.6%, and 75.7%, respectively.

Error analysis by surgical procedure type showed that long, complex procedures exhibited the highest prediction errors across all models. For the XGBoost model, the largest mean absolute errors (MAE) occurred for open reduction in spinal fracture or dislocation (*n* = 21; MAE 77.6 min), posterior thoracic fusion (*n* = 29; MAE 65.0 min), and anterior and posterior lumbar fusion procedures (MAE 47–59 min), whereas cervical and decompressive procedures showed the lowest errors (MAE 26–35 min). When stratified by the number of involved levels, errors were lowest for two-level procedures (MAE 34.3 min), intermediate for procedures with three or more levels (MAE 39.9 min) and one-level procedures (MAE 45.9 min), and highest when the level count could not be determined (MAE 59.2 min). These findings indicate that prediction error is driven more by procedure type and overall operative duration than by level count alone (Supplementary Table S4).

### 3.4. SHAP-Based Feature Importance Analysis

**Figure 2:**
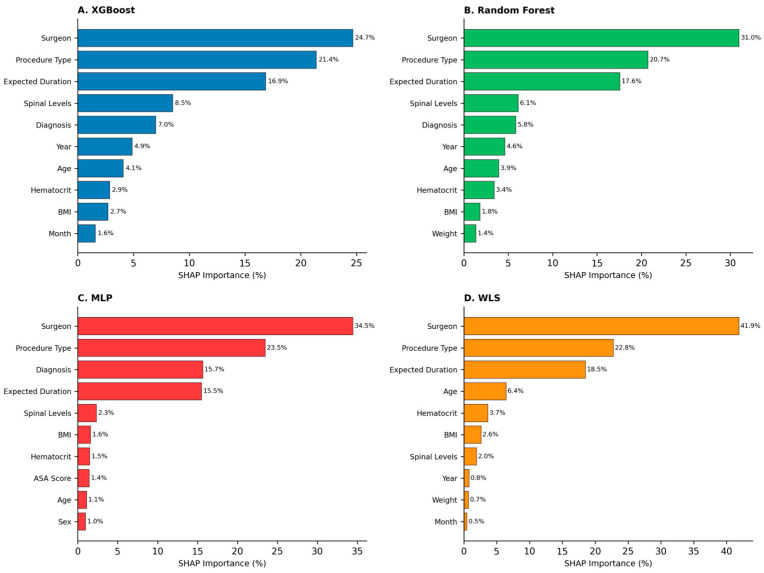
Top 10 features ranked by SHAP importance (%) for each model. (**A**) XGBoost, (**B**) Random Forest, (**C**) MLP, (**D**) WLS regression. Values represent grouped feature importance based on SHAP analysis. Grouped importance was obtained by summing the absolute SHAP values of all one-hot indicators belonging to each original variable and normalizing across variables to 100%; “duration_expected” denotes the surgeon-estimated duration.

The grouped SHAP feature importance for each model is shown in Figure 2. Surgeon identity consistently emerged as the most important feature across all four models, with relative importance ranging from 24.7% (XGBoost) to 41.9% (WLS). Surgical procedure type (Op_name) ranked second across all models (20.7–23.5%), followed by surgeon-estimated duration (duration_expected; 15.5–18.5%), which ranked third in the tree-based and WLS models. Although the surgeon’s own estimate is itself a strong predictor, its third-place ranking—behind surgeon identity and procedure type—indicates that structured, institution-specific data carry predictive information beyond the surgeon’s preoperative estimate. This ranking was robust to resampling: across repeated random subsamples of the test set, grouped importance values varied by less than ±0.5 percentage points, and surgeon identity ranked first in 100% of resamples (Supplementary Table S2).

**Figure 3:**
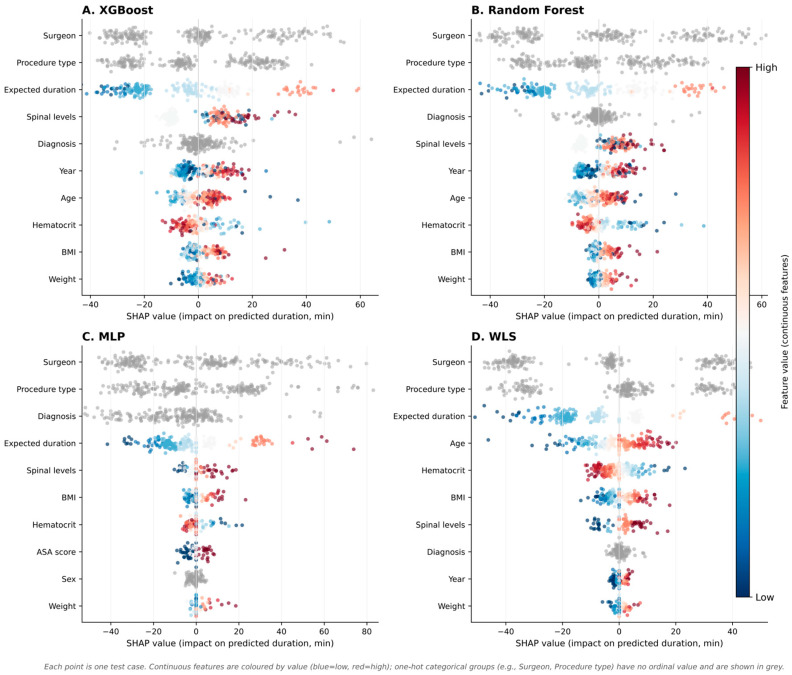
SHAP summary (beeswarm) plots illustrating the direction and magnitude of feature effects on predicted surgical duration for each model. (**A**) XGBoost, (**B**) Random Forest, (**C**) MLP, (**D**) WLS regression. Each dot represents a single prediction. Color indicates feature value (red = high, blue = low). Horizontal position indicates SHAP value (positive = increases predicted duration, negative = decreases predicted duration). SHAP values are expressed in minutes of predicted operative duration for all models.

SHAP summary plots (Figure 3) illustrate the direction and magnitude of individual feature contributions to model predictions.

Additional analyses of model stability, cross-validation fold-level performance, and sparse feature threshold sensitivity are provided in Supplementary Table S2.

## 4. Discussion

In this study, four predictive modeling approaches for spine surgery duration were compared. The XGBoost model achieved the best overall performance (R^2^ = 0.622, MSE = 3014.6 min^2^), with MLP (R^2^ = 0.621) and Random Forest (R^2^ = 0.610) performing comparably. WLS regression showed moderate accuracy (R^2^ = 0.546). Bootstrap confidence interval analysis confirmed that the three ML models performed similarly, with substantial CI overlap and MSE differences of 6.5–91.6. All three ML models outperformed WLS (MSE differences of 510.5–602.1). These results were obtained using only predictors available at the time of operating room scheduling; a full-information model that additionally included intraoperatively recorded variables performed equivalently (XGBoost R^2^ 0.622), indicating that a deployment-ready, scheduling-time model entails essentially no loss of accuracy.

The comparable performance of all three ML models carries practical implications for clinical implementation [9]. Tree-based ensemble methods inherently handle nonlinear relationships and feature interactions without explicit specification, making them well suited for complex medical datasets [8]. The MLP, despite using a fundamentally different learning paradigm (gradient-based optimization of neural network weights), achieved performance comparable to the tree-based models, suggesting that the predictive signal in this dataset is robust across different algorithmic approaches. The relative width of bootstrap confidence intervals—approximately 34% variation relative to the mean MSE for XGBoost—indicates consistent performance across different data subsets, a consideration relevant to clinical deployment. The WLS model, while showing lower accuracy than the three ML models, retains value as a transparent and computationally efficient penalized linear regression comparator.

Compared with baseline prediction using surgeon-estimated duration alone (R^2^ = 0.140, MAE = 59.8 min, on the 613 test cases with a recorded estimate), the best-performing ML models achieved a 344% increase in explained variance, an approximately 20-min reduction in MAE (paired ΔMAE −20.1 min, 95% CI −23.7 to −16.5), and a 14.0 percentage point improvement in prediction accuracy within 60 min. Even WLS regression (R^2^ = 0.546) substantially outperformed the baseline (R^2^ = 0.140). Reducing average prediction error by approximately 20 min per case represents meaningful clinical value for operating room scheduling and resource utilization [1,3].

SHAP analysis [18,19] provided clinically relevant insights into predictors of surgical duration. Surgeon identity emerged as the most influential predictor across all four models (24.7–41.9% importance), consistently outranking all other variables. Surgeon identity should be interpreted as a high-importance predictive feature rather than a direct causal driver of operative time. Beyond individual surgical technique and experience, the surgeon variable may also act as a proxy for case mix, subspecialty and procedure complexity, team and operating-room composition, institutional allocation rules, and documentation or coding practices [2,7]. Surgical procedure type ranked second (20.7–23.5%), reflecting the expected influence of procedural complexity. Surgeon-estimated duration (duration_expected), while a strong individual predictor, ranked third (15.5–18.5%), indicating that its predictive information is partially captured by the surgeon identity variable. This pattern suggests that the surgeon’s estimate functions as a composite summary that already incorporates knowledge of the procedure and patient, but the model additionally captures surgeon-specific patterns that go beyond the estimate itself. In ablation analyses, removing surgeon identity reduced XGBoost performance from an R^2^ of 0.62 to 0.54 (MAE +3.7 min), whereas removing the surgeon-estimated duration produced only a small reduction (R^2^ 0.62 to 0.60; MAE +1.6 min), confirming that the information carried by the surgeon’s estimate is largely captured by surgeon identity and procedure type (Supplementary Table S2). These findings support scheduling systems that incorporate surgeon-specific historical data rather than relying solely on the surgeon’s preoperative estimate. Beyond predictive performance, this study has important clinical implications for operating room management. More accurate prediction of surgical duration may facilitate more efficient operating room scheduling, improve allocation of personnel and resources, reduce delays and overtime, and ultimately enhance operating room utilization. These improvements may contribute to both better workflow efficiency and reduced healthcare costs.

A unique feature of the present study is the comprehensive comparison of multiple machine learning algorithms using a large real-world spine surgery dataset combined with independent test-set validation and SHAP-based model interpretability analysis. This approach not only identified the most accurate prediction model but also provided clinically meaningful insights into the factors influencing surgical duration. Methodologically, the study also pairs prediction against the operational comparator—the surgeon’s own estimate—with a leakage-controlled validation pipeline, paired bootstrap comparison of models, and a primary model restricted to information available at the time of scheduling; these elements address validation and reporting gaps that have been highlighted for machine-learning duration-prediction studies [21].

Our findings are consistent with prior work by Gabriel et al. [14] showing that ensemble machine-learning models can predict spine surgery duration. Because predictive accuracy is strongly shaped by each cohort’s case mix and duration variability, cross-study comparisons of metrics such as R^2^ are best read as contextual rather than controlled benchmarks. The present study is instead distinguished by its deployment-oriented design: a primary model restricted to information available at the time of scheduling, SHAP-based interpretation applied uniformly across all four algorithms, and paired statistical benchmarking of each model against the surgeon’s own preoperative estimate within a leakage-controlled validation framework. Recent systematic reviews have demonstrated that machine learning techniques are increasingly being applied across multiple domains of spine care, including diagnosis, outcome prediction, surgical decision-making, and healthcare resource optimization [10,11]; deep-learning methods have also been extended to image-based tasks such as automated vertebral segmentation on spine radiographs [22]. Within this broader context, the present study extends the application of machine learning to surgical duration prediction, an important yet relatively underexplored component of operating room management. Together, these findings support the growing role of artificial intelligence in improving both clinical and operational aspects of spine surgery.

Two issues raised in review warrant emphasis. First, whether an R^2^ near 0.62 is clinically sufficient depends on the use case: reducing the mean prediction error by roughly 20 min per case is operationally useful for block planning and staffing, but considerable case-to-case variability remains—particularly for long, multilevel fusions—so the model is best positioned as decision support rather than a fixed scheduling rule. Second, realizing this value requires a defined deployment pathway: in the intended workflow the model would generate a predicted duration at the time of case booking from preoperatively available inputs, which a scheduler would weigh alongside the surgeon’s estimate when allocating block time and staff. Recent multicenter and cross-specialty studies confirm that machine-learning models can outperform conventional scheduling estimates [21,23], yet evidence directly linking improved predictions to operating-room outcomes such as utilization, overtime, and turnover remains limited; generating that evidence prospectively is the necessary next step toward clinical adoption.

The improved performance of tree-based models in the present study (R^2^ = 0.622 for XGBoost) may reflect inclusion of additional variables such as the number of involved spinal levels, systematic hyperparameter optimization through cross-validation, and rigorous independent test set evaluation. The comprehensive application of SHAP analysis to all models provides a level of interpretability that facilitates clinical adoption by addressing the trust barrier that has limited deployment of ML models in health care [17,19].

Several limitations should be acknowledged. First, single-institution data may limit generalizability; multicenter validation studies are needed to confirm external validity [24]. Second, the moderate R^2^ (0.55–0.62 across models) leaves substantial surgical duration variability unexplained, with accuracy decreasing for complex, prolonged procedures involving multiple spinal levels. Additional clinical variables—including structured comorbidity indices and information extracted from unstructured operative notes via natural language processing [25]—may improve accuracy. Third, integrating predictive models into scheduling workflows requires development of intuitive user interfaces and prospective evaluation of clinical utility [26]. Fourth, the current model provides static preoperative predictions; dynamic models incorporating real-time intraoperative data may further enhance scheduling flexibility. Fifth, because model development and evaluation used a random case-level split, all nine surgeons in the test set were also represented in the training set; the reported performance therefore reflects within-institution forecasting for known surgeons and does not test generalization to new surgeons or institutions. In a temporal validation that was trained on earlier years and tested on later years, performance declined (R^2^ ≈ 0.38–0.41; MAE ≈ 48 min), indicating temporal drift in case mix and practice over the 10-year study period, although accuracy remained well above the surgeon-estimate baseline. Sixth, although the principal preprocessing steps (median imputation, one-hot encoding, sparse-feature filtering, and scaling) were fitted within the training set only, KNN imputation of a few low-missingness baseline variables was performed before the split; because removing these variables entirely did not reduce performance, any leakage transmitted through them is necessarily bounded and negligible. Future multicenter studies are warranted to validate these findings across diverse institutions and surgical settings. In addition, prospective studies evaluating the real-world implementation of machine learning-based prediction models are needed to determine their impact on operating room scheduling, resource allocation, and operational efficiency.

## 5. Conclusions

Machine learning approaches generally outperformed conventional prediction methods for spine surgery duration using only information available at the time of scheduling, with XGBoost demonstrating the highest predictive performance among the evaluated models. Surgeon identity emerged as the most influential predictor of surgical duration, highlighting the importance of surgeon-specific factors in predictive modeling. In paired comparisons, all machine learning models showed significantly lower prediction error and higher R^2^ than the surgeon-estimate baseline, whereas differences among the three best-performing models were small and not statistically significant. These findings support the potential utility of machine learning-based prediction models for operating room scheduling and resource allocation; however, because the present study evaluated predictive accuracy rather than workflow outcomes, prospective studies are needed to determine whether implementation improves operating room utilization, overtime, and scheduling efficiency.

## Figures and Tables

**Table 1:** Predictive performance of machine learning models using predictors available at the time of operating room scheduling, evaluated on the independent internal test dataset (*n* = 676).

Model	MSE (min^2^) (95% CI)	RMSE (min) (95% CI)	R^2^ (95% CI)	MAE (min)	MedAE (min)
XGBoost	3014.6 (2558.3–3556.0)	54.9 (50.6–59.6)	0.622 (0.566–0.675)	39.2	28.1
Random Forest	3106.2 (2618.9–3655.3)	55.7 (51.2–60.5)	0.610 (0.555–0.660)	39.9	29.1
MLP	3021.1 (2544.5–3536.1)	55.0 (50.4–59.5)	0.621 (0.563–0.671)	39.9	30.7
WLS	3616.7 (2984.1–4322.3)	60.1 (54.6–65.7)	0.546 (0.480–0.606)	42.4	29.0

Values are presented as point estimates with 95% confidence intervals where applicable. MSE = mean squared error; MAE = mean absolute error; MedAE = median absolute error; R^2^ = coefficient of determination; CI = confidence interval; MLP = multilayer perceptron; WLS = weighted least squares regression.

**Table 2:** Comparison of Baseline Prediction and Machine Learning Model Performance.

Metric	Baseline (Duration_Expected Only)	Best Model and Improvement
R^2^	0.140	0.622 (XGBoost)/+344% improvement
MAE (min)	59.8	39.2 (XGBoost)/34.4% reduction
RMSE (min)	83.5	54.9 (XGBoost)/34.3% reduction
Accuracy within 60 min	65.9%	80.3% (XGBoost)/+14.4 percentage points
Accuracy within 30 min	39.5%	53.6% (XGBoost)/+14.1 percentage points

Baseline prediction was calculated using surgeon-estimated operative duration (duration_expected) only, on the 613 test cases with a recorded estimate; machine-learning metrics are reported on all 676 test cases. The improvement values are direct (unpaired) differences between the tabulated baseline and best-model values; the corresponding paired estimates reported in the text (e.g., ΔMAE −20.1 min and a 14.0-percentage-point gain in accuracy within 60 min, computed on the 613 common cases) differ slightly for this reason. MAE = mean absolute error; R^2^ = coefficient of determination.

## Data Availability

The datasets used and/or analyzed during the current study are not publicly available due to institutional restrictions and patient privacy but are available from the corresponding author on reasonable request. The analysis code used for data preprocessing, model development, and evaluation is likewise available from the corresponding author on reasonable request.

## Supplementary Materials

The following supporting information can be downloaded at: https://www.mdpi.com/article/10.3390/medicina62071308/s1, Table S1. Selected final hyperparameters of each machine learning model; Table S2. Additional analyses for the primary scheduling-time model (Panels A–J); Table S3. Predictor variables used in the final models (133 features), grouped by original variable, with data source, pre-imputation missingness, and availability at the time of operating room scheduling; Table S4. Procedure-specific prediction error (XGBoost, independent test set); Figure S1. Cohort selection and data-partition flow.

## Abbreviations

ASA	American Society of Anesthesiologists
BMI	Body mass index
CI	Confidence interval
EMR	Electronic medical record
KNN	k-nearest neighbors
Lasso	Least Absolute Shrinkage and Selection Operator
MAE	Mean absolute error
MedAE	Median absolute error
ML	Machine learning
MLP	Multilayer perceptron
MSE	Mean squared error
WLS	Weighted least squares
SHAP	SHapley Additive exPlanations
