# Supplementary material for: Comparison of Machine Learning Algorithms for Predicting Spine Surgery Duration
Source: Medicina (Kaunas). 2026 Jul 6;62(7):1308. doi: 10.3390/medicina62071308 (PMC13413996; doi:10.3390/medicina62071308)
Supplement: Supplementary file 1 [file medicina-62-01308-s001.zip › Supplementary_table S3(revised_clean).pdf]

Supplementary Table S3. Predictor variables used in the final models (133 features), grouped by original variable, with data source, pre-imputation missingness, and availability at the time of operating room scheduling.

Variable (group)	Indicators	Type	Source	Missing before imputation	At scheduling
Surgeon-estimated duration	1	Numeric (min)	Surgical booking	8.1% (median-imputed)	Yes
Surgeon identity	9	One-hot	OR record	0%	Yes
Procedure type (Op_name)	13	One-hot	OR record	0%	Yes
Diagnosis	54	One-hot	Inpatient record	0%	Yes
Number of involved levels	1	Numeric	Operative plan	0%	Yes
Age / Sex	1 / 2	Numeric / one-hot	EMR	0%	Yes
Weight / Height / BMI	3	Numeric	EMR	Weight KNN-imputed (<5%)	Yes
ASA physical status	1	Numeric	Preoperative anesthesia	<5% (KNN-imputed)	Yes
Hematocrit	1	Numeric	Preoperative laboratory	<5% (KNN-imputed)	Yes
Emergency status	2	One-hot	OR record	0%	Yes
Year / Month	2	Numeric	OR record	0%	Yes
Anesthesia start time	1	Numeric (min)	Anesthesia record	0%	No (day of surgery)
Anesthesiologist	8	One-hot	Anesthesia record	KNN-imputed	No (assigned near surgery)
Anesthesia resident	27	One-hot	Anesthesia record	KNN-imputed	No
Anesthesia method	3	One-hot	Anesthesia record	0%	Partially (planned)
Anesthetic agents (sevoflurane, desflurane, O_2, remifentanil)	4	Numeric/indicator	Anesthesia record	1.2%	No (intraoperative)

Total = 133 features (15 numeric/derived variables and 118 one-hot indicators). Variables flagged “No” under “At scheduling” were excluded in the scheduling-time-only sensitivity model (Supplementary Table S2, Panel C).
