# Supplementary material for: Comparison of Machine Learning Algorithms for Predicting Spine Surgery Duration
Source: Medicina (Kaunas). 2026 Jul 6;62(7):1308. doi: 10.3390/medicina62071308 (PMC13413996; doi:10.3390/medicina62071308)
Supplement: Supplementary file 1 [file medicina-62-01308-s001.zip › Supplementary_table S4(new).pdf]

Supplementary Table S4. Procedure-specific prediction error (XGBoost, independent test set).

Panel A. Procedure groups (the five highest mean absolute error [MAE] and the lowest-error group shown).

Procedure group	n	Median actual (min)	MAE (min)	MedAE (min)	Within ±60 min
Open reduction, spinal fracture/dislocation	21	255	77.6	58.5	52.4%
Posterior thoracic fusion	29	265	65.0	54.9	55.2%
Anterior lumbar fusion	19	215	58.9	38.8	73.7%
Posterior lumbar fusion	169	230	47.3	38.2	71.0%
Posterior lumbar fusion (with cage)	33	270	43.4	28.9	81.8%
Cervical anterior fusion (lowest error)	153	135	25.7	19.3	94.1%

Panel B. Error stratified by number of involved spinal levels.

Level count	n	Median actual (min)	MAE (min)	MedAE (min)	Within ±60 min
1 level	39	240	45.9	35.2	71.8%
2 levels	351	140	34.3	23.2	85.8%
≥3 levels	219	215	39.9	31.0	77.6%
Not determinable	67	235	59.2	40.3	65.7%
