# Supplementary material for: Comparison of Machine Learning Algorithms for Predicting Spine Surgery Duration
Source: Medicina (Kaunas). 2026 Jul 6;62(7):1308. doi: 10.3390/medicina62071308 (PMC13413996; doi:10.3390/medicina62071308)
Supplement: Supplementary file 1 [file medicina-62-01308-s001.zip › Supplementary_table S1(revised_clean).pdf]

Supplementary Table S1. Selected final hyperparameters of each machine learning model.

Model	Selected final hyperparameters
XGBoost	n_estimators 250; max_depth 7; learning_rate 0.03; subsample 0.5; colsample_bytree 0.5; gamma 0.2; reg_lambda (L2) 1.0; reg_alpha (L1) 0.5
Random Forest	n_estimators 200; max_depth 50; min_samples_split 8; min_samples_leaf 1; max_features 0.3; bootstrap = True
MLP	hidden layers (100, 50); activation ReLU; optimizer Adam; alpha (L2) 0.01; learning rate constant (initial 0.001); input features standardized (StandardScaler); early stopping; remaining settings at scikit-learn defaults
WLS	Box–Cox transformation of the target ($\lambda \approx -0.10$; predictions back-transformed to minutes before scoring); 1.5×IQR residual-outlier removal (training only); Lasso (L1, $\alpha = 0.01$) feature selection (12–13 features per fold); final estimator weighted least squares (WLS) with weights = 1/(OLS residual^2 + 1e−6) to correct heteroscedasticity
