# Supplementary material for: Comparison of Machine Learning Algorithms for Predicting Spine Surgery Duration
Source: Medicina (Kaunas). 2026 Jul 6;62(7):1308. doi: 10.3390/medicina62071308 (PMC13413996; doi:10.3390/medicina62071308)
Supplement: Supplementary file 1 [file medicina-62-01308-s001.zip › Supplementary_table S2(revised_clean).pdf]

Supplementary Table S2. Additional analyses for the primary scheduling-time model (Panels A–J).

Panel A. Paired bootstrap 95% confidence intervals for differences between models on the common test cases (5,000 resamples). Negative ΔMAE/ΔRMSE and positive ΔR^2/Δaccuracy favour the first model. Comparisons with the baseline use the 613 cases with a recorded surgeon estimate.

Comparison	ΔMAE (min)	ΔRMSE (min)	ΔR^2	Δ±30 min (pp)	Δ±60 min (pp)
XGBoost − Baseline	−20.1 (−23.7, −16.5)	−27.6 (−32.0, −23.1)	+0.48 (+0.40, +0.56)	+14.0 (+9.3, +18.9)	+14.0 (+10.1, +18.3)
XGBoost − Random Forest	−0.7 (−1.8, +0.4)	−0.8 (−2.5, +0.8)	+0.01 (−0.01, +0.03)	+1.8 (−1.0, +4.4)	+0.9 (−0.9, +2.7)
XGBoost − MLP	−0.7 (−2.6, +1.2)	−0.1 (−3.1, +2.8)	+0.00 (−0.04, +0.04)	+4.9 (+1.3, +8.4)	+1.8 (−0.7, +4.4)
XGBoost − WLS	−3.2 (−5.3, −1.2)	−5.2 (−8.5, −2.2)	+0.08 (+0.03, +0.12)	+1.9 (−1.5, +5.5)	+4.6 (+1.8, +7.5)

Panel B. Feature-ablation analysis (XGBoost, independent test set).

Model variant	Features	R^2	RMSE (min)	MAE (min)
Primary model (scheduling-time)	90	0.622	54.9	39.2
− Surgeon identity	81	0.543	60.4	42.9
− Surgeon-estimated duration	89	0.601	56.4	40.8
− Both	80	0.484	64.1	46.1

Panel C. Full-information model comparison: the primary scheduling-time model (90 predictors) versus a full-information model that additionally includes intraoperatively recorded variables (anesthesia staffing, anesthesia method, anesthetic agents, and anesthesia start time; 133 predictors), on the independent test set. Performance is equivalent, confirming that restricting the model to scheduling-time predictors does not reduce accuracy.

Model	Features	R^2	RMSE (min)	MAE (min)	±60 min
Primary model (scheduling-time)	90	0.622	54.9	39.2	80.3%
Full-information model	133	0.622	54.9	39.1	79.0%

Panel D. Variable-inclusion (leakage) sensitivity for the primary scheduling-time model (XGBoost): test performance after removing the KNN-imputed baseline variables (weight, hematocrit, ASA). Removing them does not reduce performance, confirming that the pre-split KNN imputation did not materially affect the reported results. The full-information model is shown as a reference.

Variant	Features	R^2	RMSE (min)	MAE (min)
Primary model (scheduling-time)	90	0.622	54.9	39.2
Without KNN-imputed variables (leakage check)	87	0.632	54.1	38.8
Full-information model (reference)	133	0.622	54.9	39.1

Panel E. Temporal validation (train on earlier years, test on later years).

Validation scheme	Train n	Test n	R^2	RMSE (min)	MAE (min)	±60 min
Random split (primary)	2,700	676	0.622	54.9	39.2	80.3%
Train ≤2018 / test >2018	2,928	448	0.378	65.2	47.4	72.8%
Train ≤2019 / test >2019	3,143	233	0.408	68.5	49.8	67.0%

Panel F. Stability of grouped SHAP importance (XGBoost) across 50 random 300-instance subsamples; surgeon identity ranked first in 100% of bootstrap resamples.

Variable	Grouped importance, mean ± SD (%)	Range (%)
Surgeon identity	24.9 ± 0.3	24.3–25.4
Procedure type	21.4 ± 0.3	20.9–22.0
Surgeon-estimated duration	16.8 ± 0.6	15.9–17.9
Level count	8.3 ± 0.2	8.0–8.6
Diagnosis	7.1 ± 0.2	6.7–7.4

Panel G. Weighted least squares (WLS) 5-fold cross-validation on the training set (primary scheduling-time predictors). λ denotes the Box–Cox parameter; selected features are those retained by Lasso (L1, α = 0.01) within each fold.

Fold	R^2	MAE (min)	Selected features	Box–Cox λ
1	0.536	42.3	11	−0.110
2	0.503	46.3	12	−0.082
3	0.519	45.9	13	−0.090
4	0.370	52.5	11	−0.100
5	0.407	46.9	13	−0.096
Mean ± SD	0.467 ± 0.066	46.8 ± 3.3	12.0 ± 0.9	−0.096 ± 0.010

Panel H. Multilayer perceptron (MLP) 5-fold cross-validation on the training set (primary scheduling-time predictors).

Fold	R^2	MAE (min)
1	0.595	40.1
2	0.567	44.3
3	0.554	45.4
4	0.505	46.4
5	0.463	45.7
Mean ± SD	0.537 ± 0.047	44.4 ± 2.3

Panel I. Cross-validation versus independent-test performance (primary scheduling-time models). Gap = test R^2 − 5-fold mean CV R^2; small positive gaps indicate no overfitting.

Model	CV R^2 (5-fold mean)	Test R^2	Gap (ΔR^2)
XGBoost	0.582	0.622	+0.039
Random Forest	0.573	0.610	+0.037
MLP	0.537	0.621	+0.084
WLS	0.467	0.546	+0.079

Panel J. Sparse-feature threshold sensitivity (primary scheduling-time feature set; test set, n = 676). Threshold = minimum number of positive cases required to retain a binary indicator; 10 was used in the final model. Test R^2 remained within ~0.02 across thresholds, so the results do not hinge on this choice.

Min. positives to retain	Features retained	RF R^2	RF MAE (min)	XGB R^2	XGB MAE (min)
1 (≈ no filter)	305	0.606	39.7	0.608	39.9
5	116	0.613	39.4	0.622	38.9
10 (final)	85	0.612	39.6	0.622	38.9
15	66	0.609	39.8	0.625	39.2
20	61	0.606	39.9	0.618	39.2
