# Supplementary figures and images for: Comparison of Machine Learning Algorithms for Predicting Spine Surgery Duration
Source: Medicina (Kaunas). 2026 Jul 6;62(7):1308. doi: 10.3390/medicina62071308 (PMC13413996; doi:10.3390/medicina62071308)

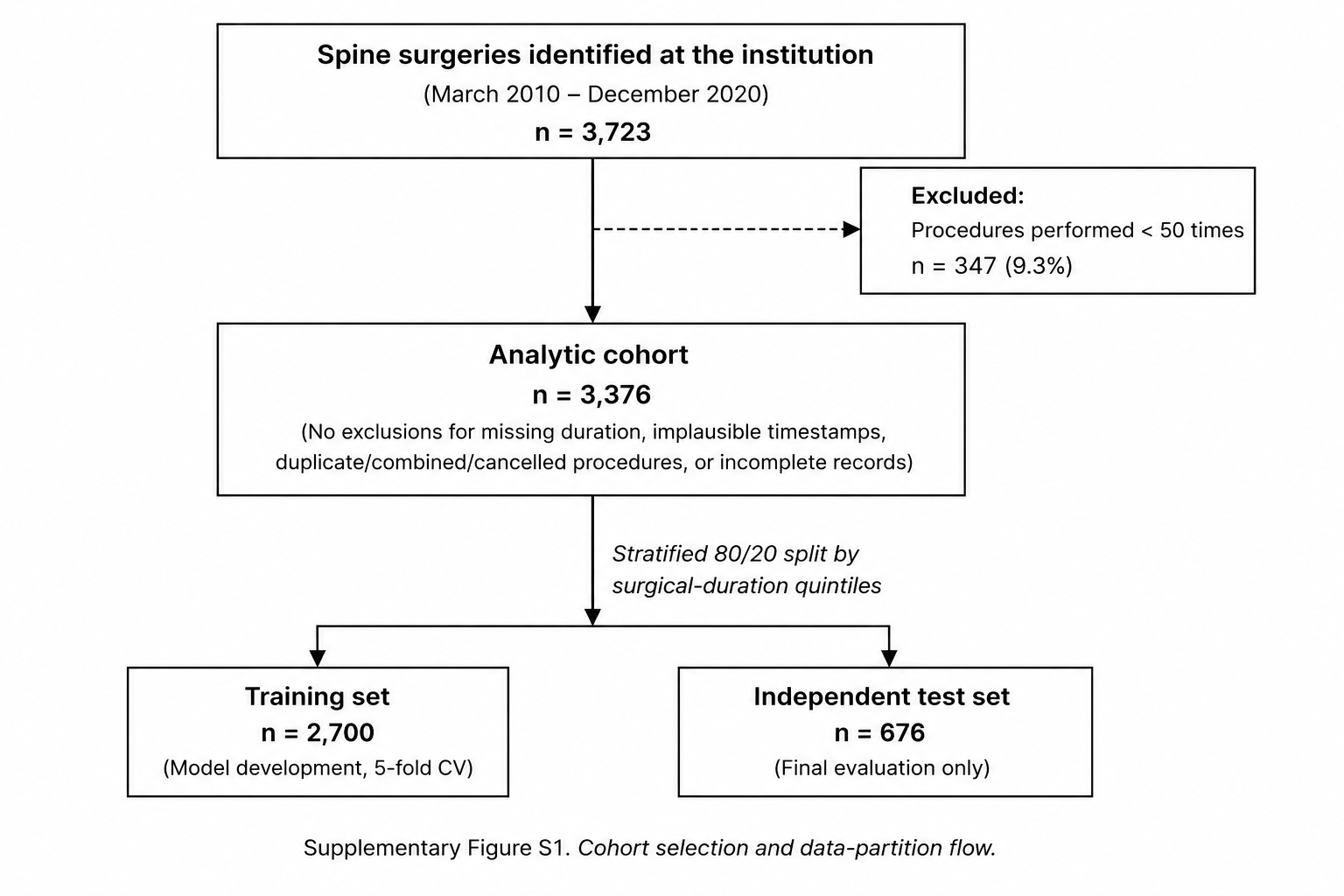

Supplement: Supplementary file 1 [file medicina-62-01308-s001.zip › supplemenatary figure S1(new).png]
